# The relationship between axial length/corneal radius of curvature ratio and stress–strain index in myopic eyeballs: Using Corvis ST tonometry

**DOI:** 10.3389/fbioe.2022.939129

**Published:** 2022-08-15

**Authors:** Zhe Chu, Qi Ren, Meizhen Chen, Lu Cheng, Hao Cheng, Wei Cui, Wenjiao Bi, Jie Wu

**Affiliations:** ^1^ Eye Institute of Shandong First Medical University, Qingdao Eye Hospital of Shandong First Medical University, State Key Laboratory Cultivation Base, Shandong Provincial Key Laboratory of Ophthalmology, School of Ophthalmology, Shandong First Medical University, Qingdao, China; ^2^ State Key Laboratory of Ophthalmology, Zhongshan Ophthalmic Center, Sun Yat-sen University Guangzhou, Guangzhou, China; ^3^ Department of Ophthalmology, the First Affiliated Hospital of Guangzhou Medical University, Guangzhou, China

**Keywords:** axial length, corneal radius of curvature, AL/CR, stress–strain index, corvis ST, sclera

## Abstract

**Purpose:** This study aimed to investigate the correlation of axial length/corneal radius of curvature ratio with stress–strain index (SSI).

**Methods:** Retrospective analysis was conducted to compare the right eyes of those with high myopia (HM, n = 132; age and 10–48 years) with those without high myopia (NHM, n = 135; age and 7–48 years), where the baseline axial length, corneal radius of curvature ratio, and central corneal thickness were analyzed; the differences in two groups were compared; and the relationship of axial length and axial length/corneal radius of curvature ratio with SSI were explored.

**Results:** Compared with AL < 26mm, SSI significantly decreased when AL ≥ 26 mm (*p* = 0.001), while there was no correlation with AL in the NHM group (*r* = -0.14, *p* = 0.12) or HM group (*r* = -0.09, *p* = 0.32). AL/CR was significantly associated with SSI in both the NHM (*r* = -0.4, *p* < 0.001) and HM (*r* = -0.18, *p* = 0.04) groups. In the NHM group, AL/CR was significantly associated with SSI (unstandardized beta = -0.514, se = 0.109, *p* < 0.001) with the adjustment of age and gender. Additionally, a significant association of SSI with AL/CR was also found after adjusting for age and gender (unstandardized beta = -0.258, se = 0.096, and *p* = 0.0082) in the HM group.

**Conclusion:** SSI showed a significant negative correlation with AL/CR in patients without high myopia and in patients with high myopia. However, SSI exhibited no decrease with the worsening of myopia, but it gradually remained stable at a low level. The findings of this study validate, to some extent, the possibility of analyzing the dynamic changes in ocular wall stiffness during the development of myopia by measuring *in vivo* corneal biomechanical parameters.

## Introduction

The pathogenesis of myopia has long been viewed as an interesting biological problem, and the development of nearsightedness is inextricably linked to the process of refractive development. Initially, this process was often described in terms of a change in the refractive error; however, it became more sophisticated with the advancement of the equipment used for examination, and we have found that even though the refractive state of the eye is maintained using orthokeratology, the structure of the eye, including the cornea and lens, and parameters such as axial length (AL) change throughout the life Morgan et al., (2014). The AL in newborns is approximately 17 mm. During the period from birth to 2–3 years of age, the corneal refractive power and lens refractive power decline rapidly, while the AL increases; after the relative stabilization of corneal development at 2–3 years of age, the AL increases rapidly, and to match this increase, the lens refractive power declines rapidly. When the rate at which the lens loses its refractive power decreases rapidly at the age of 10–12 years, the refractive state of the eye stabilizes and approaches emmetropia; at this stage, the AL is approximately 23 mm, and this process stabilization is known as emmetropization. However, after the completion of emmetropization, the increase in the AL can continue until at least 30 years of age. This condition, in which the other refractive components no longer change but the AL continues to increase, has been considered an important cause of axial myopia (Tokoro and Suzuki, 1968; Mutti et al., 2012). The increase in the AL is essentially the result of the expansion of the ocular wall. Because the sclera occupies more than 90% of the surface area of the eyeball Olsen et al., (1998), the expansion of the scleral wall is the most central change in patients with axial myopia. The wall of the eyeball is roughly shaped as a flat ellipsoid at birth, and during emmetropization, the scleral wall expands nearly uniformly in all directions, gradually changing from a flat sphere to a round sphere. However, the expansion of the wall of the eyeball is not always uniform in all directions; in normal eyes, the anterior and posterior regions of the sclera reach an adult level at 2 and 13 years of age (Fledelius and Christensen, 1996), respectively. Subsequently, as myopia develops and progresses, the expansion of the sclera is greater along the sagittal axis [0.35 mm/diopter (D)] than along the coronal axis (0.19 mm/D) and horizontal axis (0.10 mm/D), although it can continue to expand in all three axes Atchison et al., (2004). The expansion of the scleral wall on the sagittal axis (i.e., the extension of the AL length) shows the closest relation to the refractive status and prognosis of axial myopia: first, the extension of the AL drives the retina of the posterior pole back, and the degree of distance of the posterior pole retina from the focal plane of the refractive system, such as the cornea and lens, can be regarded as the degree of myopia; consequently, high myopia (HM) is defined by a spherical equivalent of −6 D or less with an AL generally exceeding 26 mm (Wang et al., 2015; Tideman et al., 2016). It should be added here that axial elongation is the primary factor in the development of myopia (Flitcroft et al., 2019; Wolffsohn et al., 2019). Considering that the AL distribution of emmetropia ranges from 21.5 to 25.5 mm Steiger, 1913), AL ≥ 26 mm (or AL ≥ 26.5 mm) is often used as the diagnostic threshold of high myopia in clinical studies. Second, especially in the patients with HM, the prevalence of posterior scleral staphyloma as well as choroidal thinning and vitreoretinal interface traction increases significantly with an increase in the AL, which leads to the impairment of visual function due to pathological myopia (Ohno-Matsui et al., 2021).

It is undeniable that AL is an extremely important parameter that reflects the degree of ocular myopia. Nevertheless, considering the differences in body mass index (BMI) and orbital volume of each individual, we can state that AL alone does not accurately reflect the degree of ocular myopia. Due to the differences in the refractive power of the cornea, lens, and other components of the refractive system between individuals, myopic refraction may vary for the same AL; therefore, the description of the degree of myopia requires the combination of the “refractive power of the refractive system” and the “distance of the photoreceptors from the focal plane of the refractive system of the eye.” The spherical equivalent refractive error is the simplest and most direct way to quantify the degree of myopia, which is described by the refractive distance between the focal plane of the refractive system of the eye and the central macular concavity. In addition, because the anterior surface of the cornea carries most of the refractive power of the refractive system of the eye, the ratio of AL and corneal radius of curvature (AL/CR) is likewise a reliable variable for the quantitative description of the degree of ocular myopia. Therefore, the AL/CR shows a better correlation with the refractive error than does AL alone (Grosvenor and Scott, 1994; Scheiman et al., 2016).

With a change in the severity of axial myopia, the biomechanical properties of the ocular wall change, and myopic eyes have been suggested to show lower levels of stiffness than do emmetropic ones (Lam et al., 2002; Berisha et al., 2010). Studies on the isolated scleral tissues have revealed that the scleral collagen fibers in highly myopic eyes show a decrease in diameter, which increases the ocular wall elasticity and viscoelasticity (Rada et al., 2006; Mcbrien et al., 2009), especially in the posterior scleral chylomicron where ultrastructural changes in the scleral tissue make the scleral structure thinner and more susceptible to mechanical stress-induced deformation (Curtin and Teng, 1958; Gentle et al., 2003). Meanwhile, studies on the biomechanical properties of the *in vivo* cornea in myopic eyes have found that the expansion of the sclera may lead to a decrease in corneal stiffness (Yu et al., 2020). Because the vast majority of the sclera is not exposed to the external environment like the cornea, few studies have focused on the biomechanical properties of the *in vivo* scleral tissue; however, some researchers believe that the above finding is because the corneal stroma is the continuation of the scleral tissue. Some corneal biomechanical parameters are related to the eye axis, and the measurement of corneal biomechanical parameters can reflect the mechanical properties of the sclera to some extent (Yu et al., 2020; Liu et al., 2021).

The CorVis ST tonometry-based stress–strain index (SSI) is a new index of corneal stiffness obtained using a numerical simulation of model eyes and finite element analysis, and it is currently considered to be age-dependent; however, it is not correlated with intraocular pressure (IOP) or central corneal thickness (CCT) (Eliasy et al., 2019). SSI is based on the stress–strain curve of the ocular wall tissue. The stress–strain curve is an important concept in material science, which can be obtained by recording the deformation (strain) of material under different tensile and compressive loading stresses (Luebkeman and Peting). With regard to the ocular wall tissue, while applying a simple stretching factor as a multiplier to all strain values, stress–strain curves that are different and have no intersecting trends could be obtained. For the average experimental behavior observed in the corneal tissue of an individual aged 50 years, SSI was set to 1.0. Higher SSI values would indicate higher tissue stiffness and vice versa (Eliasy et al., 2019). Liu et al. found that SSI, an index of corneal stiffness, was negatively correlated with AL when the latter is <26 mm but not when it is ≥26 mm (Liu et al., 2021). Therefore, it is suggested that due to the non-uniform expansion of the eyeball during myopic development, the eye tends to expand uniformly in all directions in the early stages; the expansion after the development of HM mainly originates in the posterior pole, when the morphological and mechanical properties of the anterior segment of the eye stabilize and are no longer associated with the posterior pole. These studies provide the basis for us to infer changes in the biomechanical characteristics of the myopic ocular wall using SSI.

As noted earlier, the stiffness of the ocular wall increases with age and decreases with the progression of myopia, and the performance of SSI in many studies seems to fit well with this pattern of change in the ocular wall stiffness (Eliasy et al., 2019; Liu et al., 2021). Because the AL/CR shows a better correlation with refractive error than does AL alone and because AL/CR can be used to measure the degree of ocular myopia, we wonder if SSI still fits the pattern of a decrease in wall stiffness with an increase in myopia at AL ≥ 26 mm. In other words, it is unclear whether SSI is still correlated with the expansion of the posterior pole of the eye after the morphologic and mechanical properties of the anterior segment of the eye have developed and stabilized. Therefore, we hypothesized that the biomechanical parameter SSI is not directly related to the “increase in the AL” but rather to the “increase in myopia.” To test the abovementioned hypothesis, we analyzed AL/CR and SSI as variables for the evaluation of the degree of ocular myopia and the biomechanical characteristics of the ocular wall, respectively, with the aim to explore the correlation between SSI and the degree of myopia and to investigate whether the dynamic changes in ocular wall stiffness during the development of myopia can be analyzed through the measurement of *in vivo* corneal biomechanical parameters.

## Methods

### Subjects

A total of 267 patients (534 eyes) admitted to the Qingdao Eye Hospital of Shandong First Medical University, from July 2021 to April 2022, were included in this cross-sectional study. We excluded participants with a history of or those who were suspected of contact lens use, keratoconus, and other corneal lesions and those who had undergone refractive surgery and other ophthalmic surgeries, such as vitreous surgery, and those for uveitis or glaucoma. Depending on the measured AL of the right eye, the subjects were divided into the non-HM (NHM) group (AL < 26 mm) and the HM group (AL ≥ 26 mm). To avoid the mixed influence of both eyes on the results, all the data, except for the baseline data, were obtained from the right eye. All subjects underwent a complete general ophthalmic examination, including slit lamp examination, subjective refraction measurement, fundus examination, and IOP measurement. All research procedures followed the principles of the Helsinki Declaration and were approved by the Ethics Committee of Qingdao Eye Hospital of Shandong First Medical University.

### Measurement of ocular structural parameters and biomechanical parameters

An Optical coherence biometrics OA-2000 (Tomey, Japan) was utilized to measure the AL of each subject’s eyes. Pentacam (Oculus, Wetzlar, Germany) was utilized to measure the corneal thickness and radius of curvature of the anterior corneal surface. Biomechanical SSI parameters were measured with CorVis ST (Oculus, Wetzlar, Germany). All measurements were performed by certified technicians. Only measurements with “OK” quality specifications were included in this analysis.

### Statistical analysis

Sociodemographic and clinical variables of participants were compared between the two groups (NHM vs. HM) using a two-sample *t*-test for continuous variables and Pearson’s χ2 test for categorical variables. We performed Pearson’s correlation tests to examine the relationships between continuous variables of interest in the two groups separately. We ran a multivariable linear regression model with SSI as the dependent variable for each group to investigate the association of AL/CR with SSI. The covariates included age and gender. Cook’s distance was used to detect potential influential points that may affect our models. We did not find any influential points. All statistical work was conducted using R statistical software (Version: 4.1.3).

## Result

### Sample characteristics

As shown in Table 1, the study sample comprised 267 participants, including 135 individuals in the NHM group and 132 individuals in the HM group. There were significant differences in age, SSI, CR, AL, and AL/CR between the two groups (all *p* < 0.05), while the two groups did not differ in the percentage of female gender (*χ*2 = 0.028, *p* = 0.87) or CCT (*p* > 0.05).

**TABLE 1:** Participants’ characteristics of two groups.

Characteristic	Overall, n = 267^a^	NHM, n = 135^a^	HM, n = 132^a^	*p* value^b^
Age, year	22 (8)	20 (8)	24 (7)	<0.001
Gender				0.87
Male	122 (46%)	61 (45%)	61 (46%)	
Female	145 (54%)	74 (55%)	71 (54%)	
SSI (OD)	0.82 (0.15)	0.85 (0.16)	0.79 (0.15)	0.002
SSI (OS)	0.84 (0.15)	0.86 (0.15)	0.82 (0.15)	0.017
CCT (OD), mm	544 (34)	542 (36)	545 (32)	0.5
CCT (OS), mm	544 (34)	543 (35)	545 (33)	0.6
CR (OD), mm	7.78 (0.24)	7.71 (0.24)	7.86 (0.22)	<0.001
CR (OS), mm	7.76 (0.30)	7.68 (0.33)	7.85 (0.24)	<0.001
AL (OD), mm	26.01 (1.48)	24.89 (0.85)	27.16 (1.06)	<0.001
AL (OS), mm	25.88 (1.48)	24.82 (0.88)	26.96 (1.15)	<0.001
AL/CR (OD)	3.34 (0.17)	3.23 (0.13)	3.46 (0.14)	<0.001
AL/CR (OS)	3.34 (0.21)	3.24 (0.17)	3.44 (0.19)	<0.001

Abbreviations: NHM, non-high myopia; HM, high myopia; SSI, stress–strain index; CCT, central corneal thickness; CR, corneal curvature; AL, axial length; AL/CR, axial length/corneal radius of curvature ratio.

a Mean (SD); n (%).

b Welch two-sample *t*-test; Pearson’s Chi-squared test.

### Correlation between several continuous variables of interest

**FIGURE 1:**
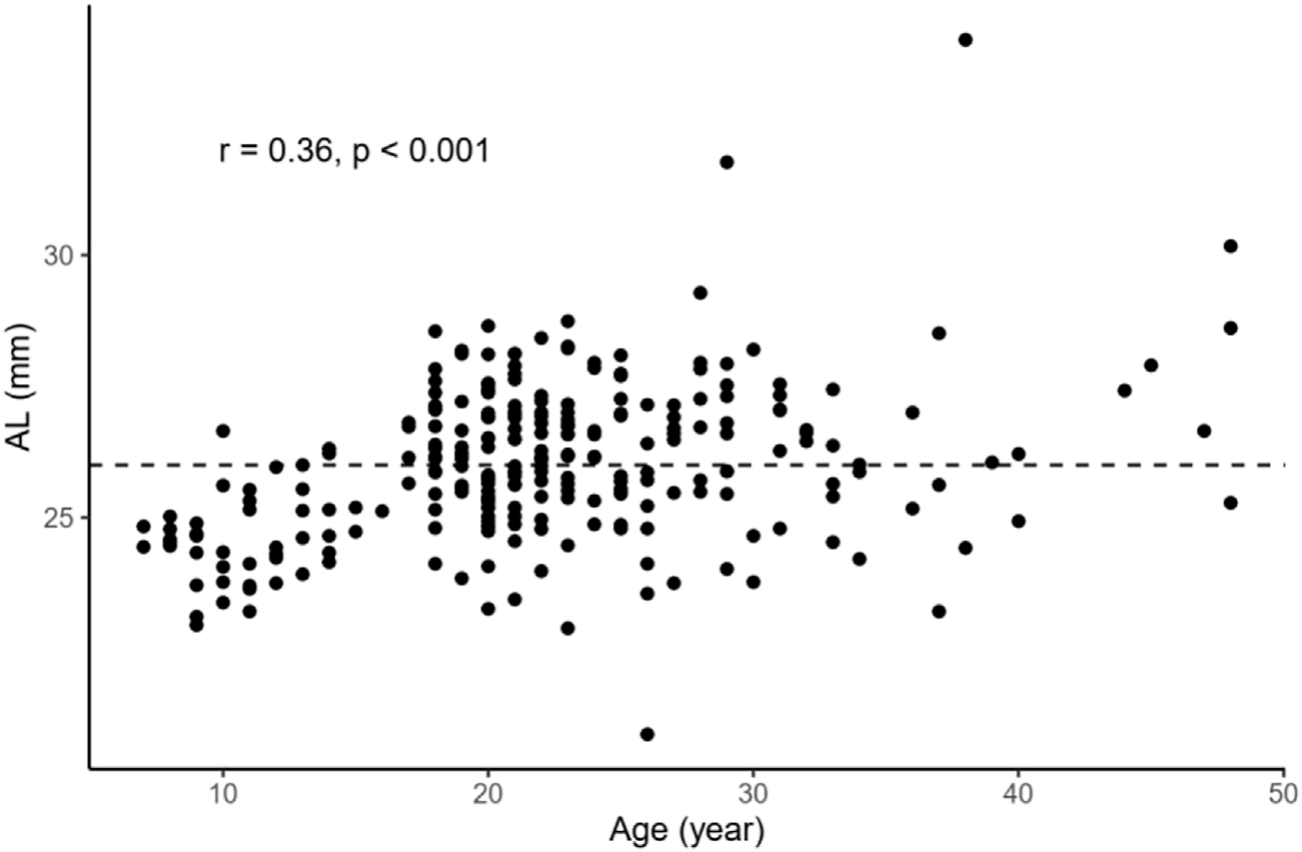
Relationship between age and AL in the overall sample. Age was positively correlated with AL in the overall sample (*r* = 0.36, *p* < 0.001). The dashed line represents the cutoff point for classifying individuals into the two groups: NHM (AL<26 mm) and HM(AL≥26 mm). Abbreviation: NHM, non-high myopia; HM, high myopia; AL, axial length.

**FIGURE 2:**
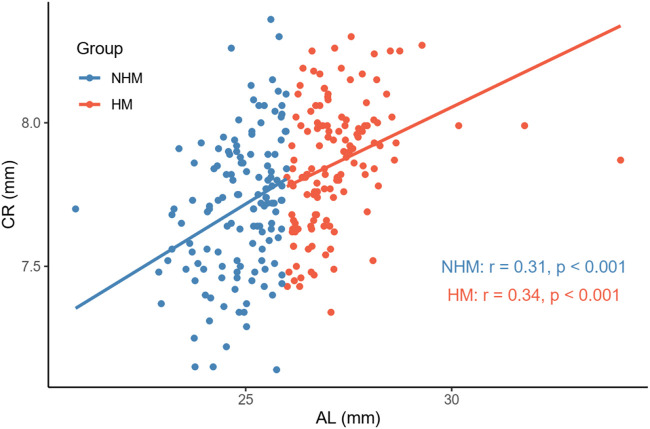
Relationship between AL and CR in the two groups. There was a positive correlation between AL and CR in both the NHM (*r* = 0.31, *p* < 0.001) and HM (*r* = 0.34, *p* < 0.001) groups. Abbreviation: NHM, non-high myopia; HM, high myopia; AL, axial length; CR, corneal curvature.

**FIGURE 3:**
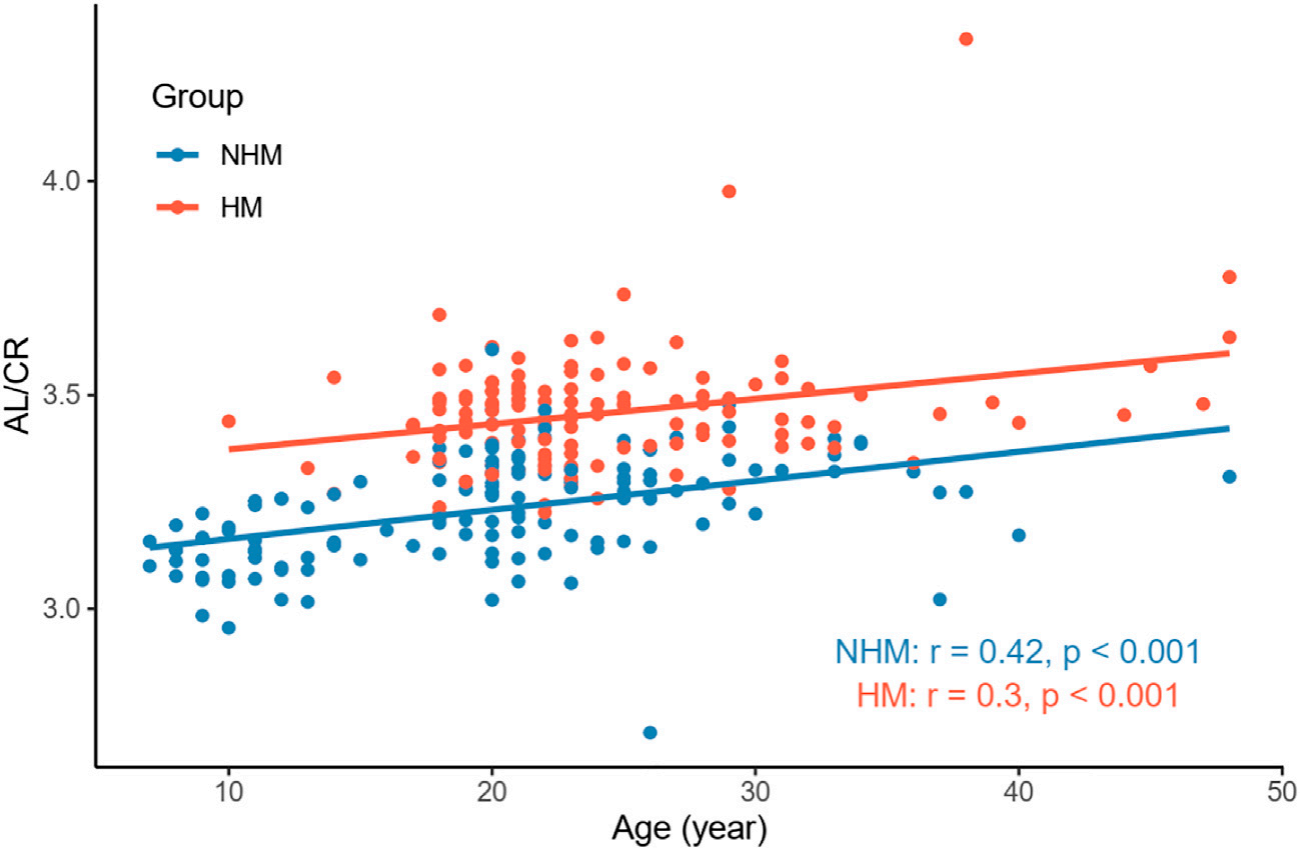
Relationship between age and AL/CR in the two groups. Age was associated with AL/CR in both the NHM (*r* = 0.42, *p* < 0.001) and HM (*r* = 0.3, *p* < 0.001) groups. Abbreviation: NHM, non-high myopia; HM, high myopia; AL/CR: axial length/corneal radius of curvature ratio.

In order to examine the relationships between several continuous variables of interest, Pearson’s correlation tests were performed. As shown in Figure 1, we found that age was positively correlated with AL in the overall sample (*r* = 0.36, *p* < 0.001). Figure 2 demonstrates a positive correlation between AL and CR in both the NHM (*r* = 0.31, *p* < 0.001) and HM (*r* = 0.34, *p* < 0.001) groups. Additionally, age was also found to be associated with AL/CR in both the NHM (*r* = 0.42, *p* < 0.001) and HM (*r* = 0.3, *p* < 0.001) groups (Figure 3).

**FIGURE 4:**
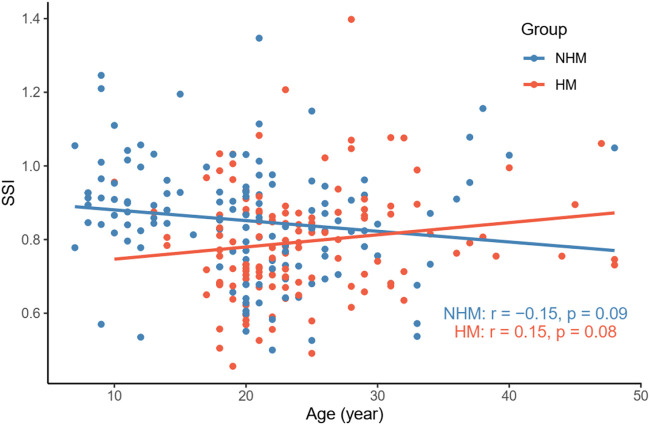
Relationship between age and SSI in the two groups. We did not find a significant correlation between age and SSI in the NHM (*r* = -0.15, *p* = 0.09) or HM (*r* = 0.15, *p* = 0.08) group. Abbreviation: NHM, non-high myopia; HM, high myopia; SSI, stress–strain index.

**FIGURE 5:**
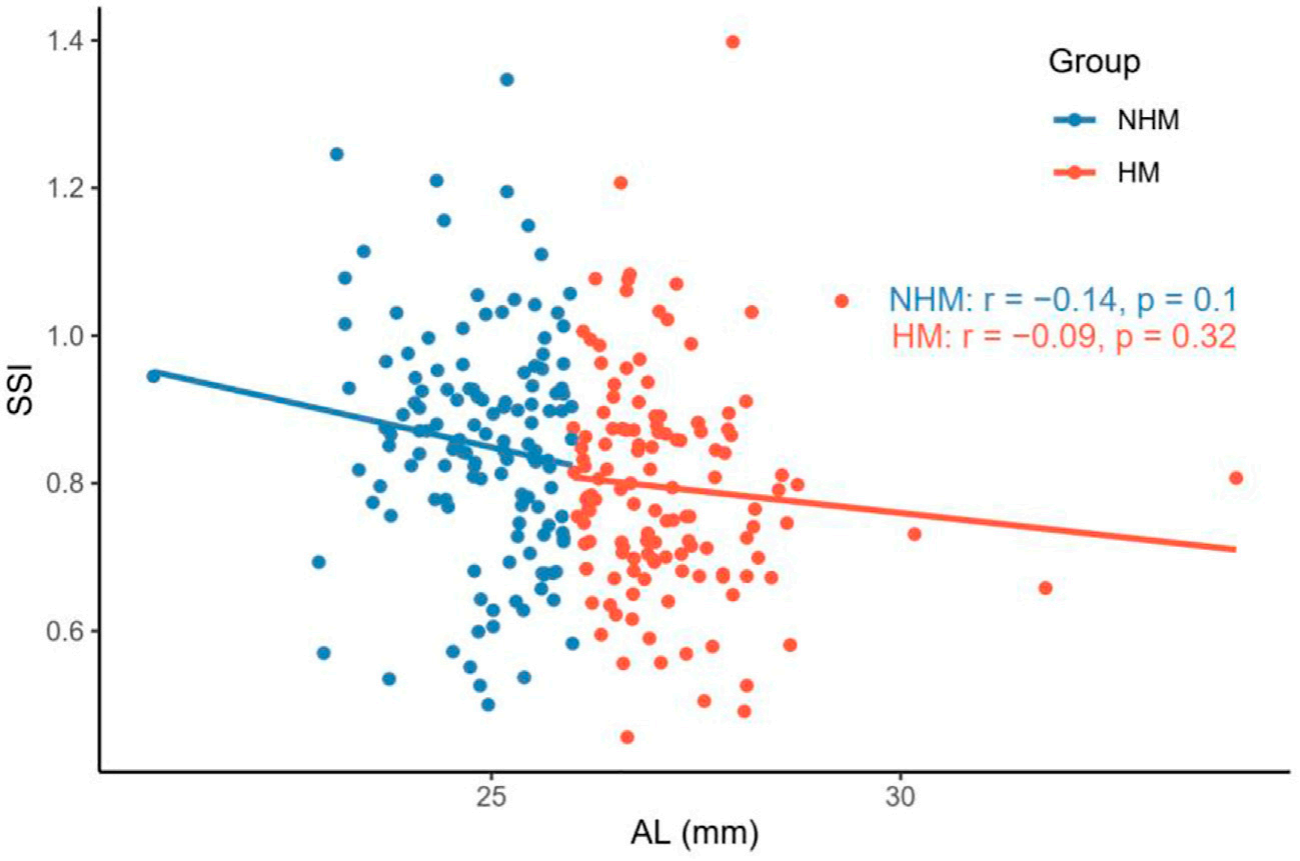
Relationship between AL and SSI in the two groups. AL was not associated with SSI in the NHM or HM group. Abbreviation: NHM, non-high myopia; HM, high-myopia; SSI, stress–strain index; AL, axial length.

Nevertheless, we did not find a significant correlation between age and SSI in the NHM (*r* = -0.15, *p* = 0.09) or HM (*r* = 0.15, *p* = 0.08) group (Figure 4). As shown in Figure 5, AL was not associated with SSI in the NHM (*r* = -0.14, *p* = 0.1) or HM group (*r* = -0.09, *p* = 0.32).

**FIGURE 6:**
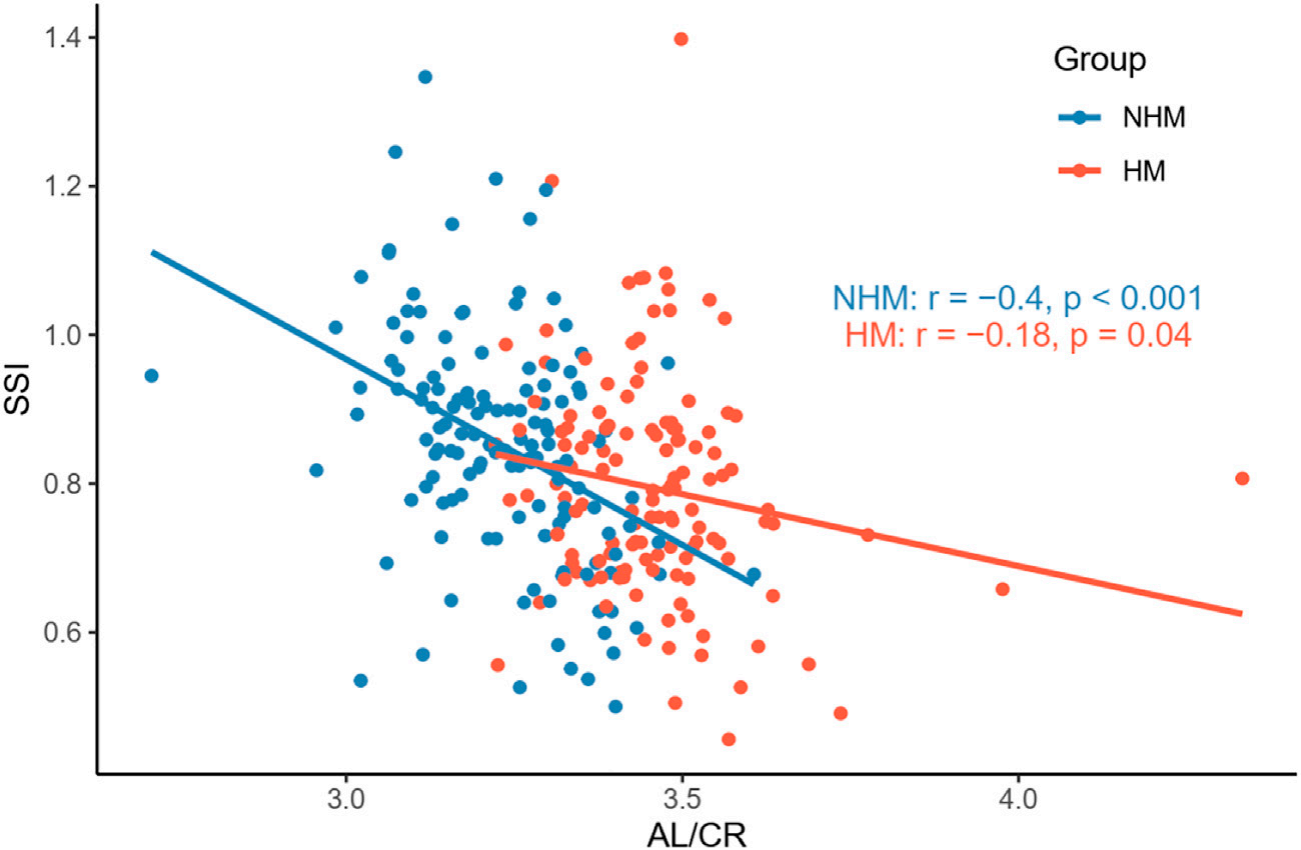
Relationship between AL/CR and SSI in the two groups. AL/CR was significantly associated with SSI in both the NHM and HM groups. Abbreviation: NHM, non-high myopia; HM, high myopia; SSI, stress–strain index; AL/CR: axial length/corneal radius of curvature ratio.

To investigate the relationship between AL/CR and SSI in the two groups, Pearson’s correlation tests were used. We found that AL/CR was significantly associated with SSI in both the NHM (*r* = -0.4, *p* < 0.001) and HM (*r* = -0.18, *p* = 0.04) groups (Figure 6).

### Association of AL/CR with SSI in the NHM and HM groups

To further explore the association between AL/CR and SSI, multivariable linear regression models with the adjustment of age and gender were fitted in the two groups. In the NHM group, AL/CR was significantly associated with SSI (unstandardized beta = -0.514, se = 0.109, *p* < 0.001) with the adjustment of age and gender (Table 2). Additionally, a significant association of SSI with AL/CR was also found after adjusting for age and gender (unstandardized beta = -0.258, se = 0.096, *p* = 0.0082) in the HM group.

**TABLE 2:** Modeling of the potential association of SSI with AL/CR adjusted for age and gender.

	NHM				HM			
	Beta	se	*t* value	*p* value	Beta	se	*t* value	*p* value
Age	0.001	0.002	0.394	0.6944	0.005	0.002	2.654	0.009
Female gender	-0.026	0.025	-1.065	0.2888	0.036	0.025	1.463	0.1458
AL/CR	-0.514	0.109	-4.728	<0.001	-0.258	0.096	-2.687	0.0082

AL/CR: axial length/corneal radius of curvature ratio; NHM, non-high myopia; HM, high myopia.

Beta represents unstandardized beta; se represents standard error; R-squared of the regression model for the NHM group is 0.17; R-squared of the regression model for the HM group is 0.09.

## Discussion

In this study, we investigated the relationship between SSI and AL/CR, a proxy parameter for the degree of ocular myopia, in the young and middle-aged populations. Overall, SSI at AL ≥ 26 mm was smaller than that at AL < 26 mm. Both at AL < 26 mm and at AL ≥ 26 mm, AL/CR values were significantly associated with SSI after the adjustment of age and gender; however, the decrease in SSI at AL ≥ 26 mm (HM group) was lower than that at AL < 26 mm (NHM group) for each unit of increase in AL/CR.

The SSI derived using CorVis ST Tonometry is intended to be independent of IOP and corneal geometry and can visually quantify corneal tissue stiffness: the greater the SSI value, the greater the stiffness of the tissue material and vice versa. In this study, SSI in the HM group was 0.77 ± 0.15 (range, 0.46–1.40), and that in the NHM group was 0.85 ± 0.16 (range, 0.50–1.35); SSI values in both the groups conformed to normal distribution. One study (Liu, 2020) reported that the SSI values in a Chinese Han cohort aged 17–50 years (mean, 27.4 years old) showed a normal distribution with a mean value of 0.895 (range, 1.16–0.57). This finding was consistent with the results of the current study (Table 1).

It has been suggested that as glycation-induced cross-linking increases with age, corneal stiffness increases (Daxer et al., 1998; Elsheikh et al., 2010). Moreover, due to increased non-enzymatic glycosylation and cross-linking of collagen fibers, the stiffness of the sclera increases with age (Schultz et al., 2008; Coudrillier et al., 2012) [stiffnesses of the anterior and posterior sclerae vary depending on the developmental cycle (Spaide, 2014)]. Thus, the corneal stroma, as a continuation of the sclera, can reflect the biomechanical properties of the sclera to some extent. In a large multicenter clinical trial (Eliasy et al., 2019) (mean age of participants, 40.6 ± 17.1 years; range, 7–87 years), SSI that was measured *in vivo* was demonstrated to increase with age, and this positive correlation between SSI and age was later reported by Liu et al. to be independent of AL (Liu et al., 2021). This relationship between SSI and age was not well reproduced in the current study (Figure 4). One study (Liu et al., 2020) found that SSI was relatively stable before the age of 35 and then increased significantly with age; thus, the relative concentration of participant age in the current study (mean age, 22.1 ± 7.7 years; range, 7–48 years) may explain the contradictory results (Table 1).

A recent study reported a negative correlation between SSI and AL when the latter was <26 mm; this correlation did not exist when AL was ≥26 mm. Liu et al. speculated that this result may be explained by the non-uniform expansion of the eye during the development of myopia: during the early stages of myopia development, the eyeball tends to dilate uniformly in all directions, whereas after the development of HM, the dilatation mainly originates in the posterior pole, and the morphological and mechanical properties of the anterior segment of the eye stabilize and are no longer relevant to the posterior pole, which explains this result (Liu et al., 2021). The current study also showed that SSI was significantly lower in the eyes with AL ≥ 26 mm than in eyes with AL < 26 mm, but the correlation between AL and SSI did not reach a statistically significant level in any of the groups (Figure 5). Therefore, it seems that changes in the biomechanical properties in the ocular wall of the HM group are not directly related to the “increase in the AL.” Some studies have shown that the cornea may play an emmetropizing role in preserving emmetropia or low myopia. This emmetropizing capacity could be insufficient when the AL is excessive in the ocular globe; this insufficiency leads to the appearance of myopia (González Blanco et al., 2008). The reason why the negative correlation between SSI and AL is no longer apparent at AL ≥ 26 mm may be related to the stabilization of CR. Moreover, considering the heterogeneity of the ocular wall expansion, perhaps we may obtain a more accurate relationship between SSI and AL by stratifying the eyeball morphology for the same AL after introducing ocular sagittal and coronal diameter variables, which warrants future validation with larger sample sizes.

According to previous studies, AL/CR is more closely related to myopia than AL alone (Fledelius, 1986; Ojaimi et al., 2005; Foo et al., 2016; Scheiman et al., 2016; Jong et al., 2018), and the stronger correlation between AL/CR and refractive error indicates the dynamic balance between AL and the corneal curvature during the development of myopia (Ojaimi et al., 2005). On the basis of this finding, the current study used AL/CR, the variable that is more closely related to the degree of myopia, to investigate the relationship between SSI and HM. First, in the whole sample, age was positively correlated with AL (Figure 1). AL and CR were positively correlated in both the NHM and HM groups (Figure 2). In addition, age was also correlated with AL/CR in the NHM and HM groups (Figure 3). To further explore the relationship between AL/CR and SSI, age- and sex-adjusted multiple linear regression models were fitted for both the groups. First of all, just as myopia is not necessarily more severe at AL ≥ 26 mm than at AL < 26 mm, NHM and HM in this study showed a wide intersection in the interval of approximately 3.2–3.6 on the AL/CR coordinate axis of the function (Figure 6). Meanwhile, as predicted, SSI after adjusting for age and sex showed a significant negative correlation with AL/CR in the NHM group and the HM group (Table 2), but the decrease in SSI was lesser with each unit of increase in AL/CR in the HM group than in the NHM group, indicating that SSI does not keep decreasing with an increase in myopia, but it gradually stabilizes at a lower level. This suggests that the insignificant correlation between SSI and AL at AL ≥ 26 mm may be related to the stabilization of the biomechanical properties of the cornea as well.

This study focuses on the correlation between the corneal biomechanical index SSI and the variable AL/CR, which reflects the degree of myopia, in the population with HM and NHM. We found that if AL/CR is used as the variable to measure the degree of myopia, the SSI still fits the pattern of a decrease with an increase in myopia at AL ≥ 26 mm, which indicates that the SSI is still correlated with the expansion of the posterior pole of the sclera after the morphological and mechanical properties of the anterior segment of the eye have developed and stabilized. However, with regard to the research methods, some limitations need to be acknowledged. First, although we verified the hypothesis that SSI is more strongly related to the degree of myopia than is the increase in the AL, the causal relationship between changes in the biomechanical properties of the ocular wall and changes in the growth of the eye axis cannot be determined because the present study is a cross-sectional study. Second, the results are based on variables associated with axial myopia, and it is not known whether refractive myopia applies to these results due to the lack of “refractive errorˮ in the variables. In addition, it is also difficult to accurately interpret the SSI values measured under the *in vivo* conditions, where the eyeball is suspended in the soft tissue and the posterior sclera. Although the posterior sclera is relatively less rigid, its extension by the support of the retrobulbar tissue itself is limited; the anterior sclera, although more rigid, protrudes mostly outside the orbit and is not supported by the retrobulbar tissue. Therefore, it is difficult to determine the extent to which the anterior and posterior scleral strain contributes to the SSI values (Mcbrien et al., 2009; Girard et al., 2011), although this does not seem to affect the assessment of the overall biomechanical characteristics of the eye *in vivo* using SSI. Further, the findings of this study also validate, to some extent, the possibility of analyzing the dynamic changes in ocular wall stiffness during the development of myopia by measuring *in vivo* corneal biomechanical parameters.

As myopia research continues to advance, our understanding of the mechanisms behind the development of myopia is evolving. However, so far, we do not know the answers to the fundamental questions on the nature of myopia and the reasons for its occurrence and progress. With the development of a series of *in vivo* imaging techniques such as swept-source optical coherence tomography and 3D-MR, our understanding of myopia has evolved from the initial one based on refractive changes and morphological changes in the eye to the present-day *in vivo*, dynamic regulation mechanisms. On this basis, the present study also provides new ideas for us to investigate the effects of myopia on the biomechanical characteristics of the eyeball under *in vivo* conditions.

## Conclusion

SSI showed a significant negative correlation with AL/CR in patients without high myopia and in patients with high myopia. However, SSI exhibited no decrease with the worsening of myopia, but it gradually remained stable at a low level. The findings of this study validate, to some extent, the possibility of analyzing the dynamic changes in ocular wall stiffness during the development of myopia by measuring *in vivo* corneal biomechanical parameters.

## Data Availability

The raw data supporting the conclusion of this article will be made available by the authors, without undue reservation.
